# Artificial Neural Network-Based Uplink Power Prediction From Multi-Floor Indoor Measurement Campaigns in 4G Networks

**DOI:** 10.3389/fpubh.2021.777798

**Published:** 2021-11-30

**Authors:** Taghrid Mazloum, Shanshan Wang, Maryem Hamdi, Biruk Ashenafi Mulugeta, Joe Wiart

**Affiliations:** Chaire C2M, LTCI, Télécom Paris, Institut Polytechnique de Paris, Palaiseau, France

**Keywords:** EMF exposure, indoor, uplink, LTE, transmit power, artificial neural networks

## Abstract

Paving the path toward the fifth generation (5G) of wireless networks with a huge increase in the number of user equipment has strengthened public concerns on human exposure to radio-frequency electromagnetic fields (RF EMFs). This requires an assessment and monitoring of RF EMF exposure, in an almost continuous way. Particular interest goes to the uplink (UL) exposure, assessed through the transmission power of the mobile phone, due to its close proximity to the human body. However, the UL transmit (TX) power is not provided by the off-the-shelf modem and RF devices. In this context, we first conduct measurement campaigns in a multi-floor indoor environment using a drive test solution to record both downlink (DL) and UL connection parameters for Long Term Evolution (LTE) networks. Several usage services (including WhatsApp voice calls, WhatsApp video calls, and file uploading) are investigated in the measurement campaigns. Then, we propose an artificial neural network (ANN) model to estimate the UL TX power, by exploiting easily available parameters such as the DL connection indicators and the information related to an indoor environment. With those easy-accessed input features, the proposed ANN model is able to obtain an accurate estimation of UL TX power with a mean absolute error (MAE) of 1.487 dB.

## 1. Introduction

Human exposure to radiofrequency electromagnetic field (RF-EMF) has been addressed and monitored over the years, especially with the succession of generations of cellular networks, the massive deployment of base stations, and the exponential increase in the number of RF devices (including connected objects of the Internet of Things IoT). Such monitoring aims to verify RF-EMF compliance with international guidelines such as the ones recommended by the International Commission on Non-Ionizing Radiation Protection (ICNIRP) (1) in order to reply to public concerns on the health impact of RF-EMF exposure. The characterization of human exposure to RF-EMF could be performed by carrying out measurement campaigns (2) and simulations (3). A survey on RF-EMF exposure in indoor environments is provided in (4).

There are several ways to measure RF-EMF exposure. Downlink (DL) exposure induced by outdoor base stations (5) or indoor access points/femtocells (6) could be assessed by measuring the electric field strength using a spectrum analyzer. Moreover, uplink (UL) exposure induced by a user equipment (UE), together with the DL exposure, could be evaluated by using network-based tools that allow recording a huge amount of data related to, e.g., number of connected UEs, UE transmit (TX) power, UE received power, and throughput (7–11). However, these data are just accessible to the network operator. Nevertheless, the UL exposure could also be assessed using mobile-phone based tools, such as a drive test solution (12, 13), and android-based applications, such as XMobiSense (14, 15). The former enables recording network information of air interface and mobile application quality-of-service and quality-of-experience, such as the Nemo Handy from KeySight Technologies (16). While drive test solutions are very expensive and not accessible for the public to perform daily personal measurements, android-based applications (e.g., XMobiSense) allow measuring several parameters but not the TX power nor throughput. Nonetheless, a specific equipment (i.e., OPTis-P8E, Innowireless Co., Ltd.) with a control software is also used for the recording of both DL and UL EMF exposure, even in fifth generation (5G) new radio environment (17, 18).

Recently, machine learning (ML) and artificial neural networks (ANNs) are intensively applied in the 5G cellular networks and beyond. The potential of ML and ANN are also being investigated in the field of RF propagation and human exposure to RF-EMF. From the DL point of view, the exposure map of the 14*th* district of Paris was built using a hybrid connected ANN, which is trained with both simulated drive test and sensor network measurements (19). In (20), a simple feed forward ANN was proposed for multi-source indoor WiFi scenarios, where three access points and several WiFi clients were distributed in the floor layout of the building. Both DL and UL exposure were evaluated by feeding the ANN model with position and type of WiFi sources, position and material characteristics of walls. The collection of data about the electric field strength (i.e., the ground truth) was performed through simulations according to a deterministic method. In (21), the UL exposure due to 4G connections is assessed by predicting the UE TX power using three different ML algorithms and by changing the set of input parameters. The ground truth was obtained through conducting measurement campaigns while driving a car in Germany. Different from an outdoor scenario, the indoor environment is much more complicated due to the existence of multi walls, multi floors, furniture and their different penetration losses. The challenges are how to incorporate the uncertainty caused by real-life indoor measurements and how to extract key features that affect UE TX power from measured network parameters as well as the indoor environments.

In this paper, we propose a feed-forward ANN model that allows predicting the UE TX power, while training the model with measurement data in an indoor environment. The Nemo Handy is used to collect data on each floor of a residential building, while it is connected to a 4G outdoor base station. Several usage services were scheduled on the Nemo Handy: WhatsApp voice calls, WhatsApp video calls, and file uploading to Dropbox. The possible parameters from the DL connection indicators and their correlation with TX power are analyzed. Then, the proposed prediction model is fed with the most influential and easily available input parameters that are recorded by the Nemo Handy, but also could be available from android-based mobile phone applications. Such input parameters reveal information about DL network connection, in addition to other parameters related to the environment. The difficulty remains in using measurement data instead of simulated data since we are not able to control the measurement results. Indeed, we do not control and manage the Nemo Handy since we do not know exactly how the recorded values are computed, as explained in section 2. Consequently, we tested different averaging duration of the measurement data to find a balance of removing noise of measurement and keep enough amount of measurement data. The performance of ANN models from the averaging duration of measurement data over 1, 3, and 5 s are compared. The results show averaging measurement data for 5 s can provide the best prediction accuracy with a mean absolute error (MAE) of 1.487 dB.

To the author's knowledge and according to the literature, very rare works investigate ML and ANN methods for the prediction of the LTE UE TX power from real empirical data. Accordingly, the main contributions of this work are the following:

1. The proposal of drive test measurement protocol considering various usage services over LTE connections, including WhatsApp voice calls, WhatsApp video calls, and file uploading.
2. The proposal and performing of multi-floor indoor measurement campaigns of both DL and UL LTE connections.
3. The proposal and validation of a simple feed-forward ANN model with easy-accessed input feature to predict LTE UE TX power from indoor empirical data.

The paper is organized as follows: section 2 presents the material used and describes the measurement and the data collection. Section 3 explains the proposed ANN model that is used to predict the UE TX power over 4G connections. Section 4 presents the results of the measurement analysis as well as the TX power estimation of the ANN model. Section 5 discusses and compares the results with related works. It also presents some future works. Section 6 gives the conclusion.

## 2. Materials and Measurement Description

### 2.1. UL Power Control Mechanism

The UE TX power *P*_*TX*_ (in dBm) is set through a power control algorithm according to the 3rd Generation Partnership Project (3GPP) LTE specification 36.213, as follows (22):


(1) $$\begin{array}{r} P_{TX}=min\left\{P_{max},P_{0}+10Log_{10}\left(M\right)+\alpha PL+\Delta_{MCS}+\delta \right\}, \end{array}$$

where

- *P*_*max*_ is the maximum allowed TX power, which is equal to 23 dBm for class 3 UE.
- *P*_0_ is a cell specific parameter that represents the requested signal to interference and noise ratio per physical resource block for the reception at the base station side.
- *M* is the number of physical resource blocks allocated to the UE. It depends on the UE usage service and the cell traffic load.
- α is a cell specific parameter representing the path loss compensation factor.
- *PL* is the DL path loss estimated by the UE based on the reference signal received power (RSRP) or the received signal strength indicator (RSSI).
- Δ_*MCS*_ is a specific modulation and coding scheme factor.
- δ is a closed loop correction value that aims to compensate the fast fading variation.

### 2.2. Measurement Description and Protocol

**Figure 1:**
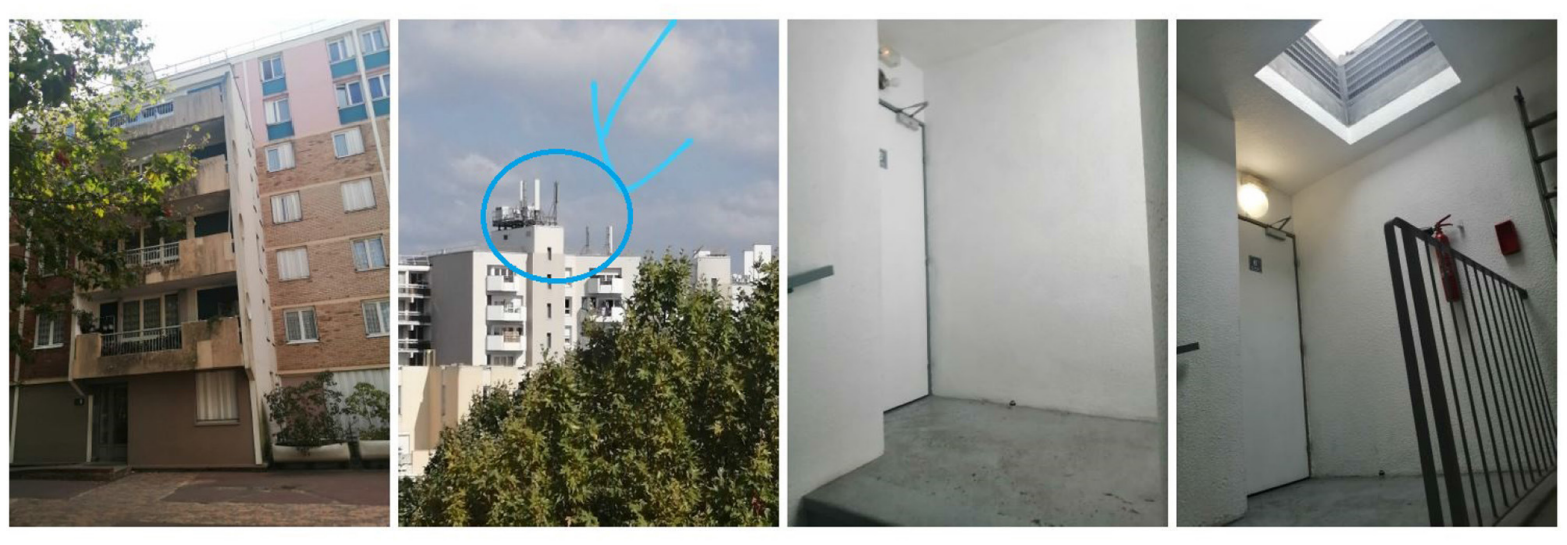
Measurement environment. From **left** to **right**: Front look, Base station, Floor 5, Floor 6.

We carried out measurement campaigns in a 6-floor residential building in May and August 2021. At each floor level (from ground floor to floor 6), one measurement location is considered, which corresponds to almost the same relative position in corridor of the stairs. The measurements were repeated 3 times over the journey: in the morning (10–11 a.m.), at noon (12–2 p.m.), and in the afternoon (4–5 p.m.), in order to take into consideration the time-varying traffic of the connected base station. We note that the measurements at night, which have the lowest traffic load (23), were not performed due to the requirement of human intervention. Figure 1 shows the measurement environment. The residence from outside and the closest base station are, respectively, shown in the left two figures. The location of measurement inside the building on floors 5 and 6 are, respectively, shown in the right two figures. The floor plans from floor 1 to 5 are identical. It is noteworthy that the residence is very close to a base station. We aim to conduct the measurements at different floors to take into account the impact of the elevation plane and the elevation angle of the base station antenna. In other words, how does the UL exposure vary with respect to the floor level?

We focus in this work on predicting the UE UL TX power while connected to 4G networks. To this end, we need to gather data about the UE UL power as well as other parameters influencing it (as explained in section 3.1). The UE UL power is recorded using dedicated drive test mobile phone solutions. Accordingly, the Nemo Handy from KeySight Technologies (16) is used in our measurement campaigns in order to log data about the UE TX power and other network parameters. The Nemo Handy is equipped on a Samsung Galaxy S20+ 5G. It supports frequency bands from 2G to 5G NR networks (up to 40 GHz).

Moreover, Nemo Handy allows scheduling certain usage services by creating scripts. Indeed, it records network parameters and saves log files while running these scripts. This is crucial because the UE UL power depends on the usage service. In our measurement campaigns, we lock the Nemo Handy to 4G networks, without locking to a given frequency band. The Nemo Handy is scheduled to run automatically the following usage services:

1. WhatsApp voice call: It is a voice over IP (VoIP) service. It is performed by emitting a voice using a speaker. The voice is reading a well-formatted text with almost 50% silence. The duration of the voice call is 2 min.
2. WhatsApp video call: The same settings are considered for the WhatsApp voice call.
3. Data upload: A 200-MB file is uploaded to Dropbox. The duration of uploading such a file depends on the network quality. In our case, it varies between almost 3 and 12 min.

We note that we mimic a realistic usage scenario during the measurements. The mobile phone was held by the experimenter at a height of about 1 m.

After carrying out the measurements, we use the Nemo outdoor software (installed on a laptop) in order to extract from the log files several parameters, mainly the following data: the UE TX power, the RSRP, and RSSI. All these powers were recorded in dBm. Moreover, we extract the physical cell identity (PCI) of the connected base station in order to check if the active base station remains the same in all the measurements. We are not able to locate the connected base station since its PCI is not accessible to public. It is just known by the network operator. Furthermore, we note that Nemo sampling method of the recorded data is unknown. The number of samples per second is not constant, it changes between 0 and 5 samples per second. Indeed, it is not possible to know how the Nemo computes the recorded values: if they are instantaneous values, averaged values over how the long sample period, etc. Therefore, we decided to compute average values over different time periods (i.e., 1, 3, and 5 s) and compare the corresponding performance. The processing and analysis of extracted parameters, as well as building ANN using them, are provided in section 4.

## 3. ANN Model for TX Power Estimation

**Figure 2:**
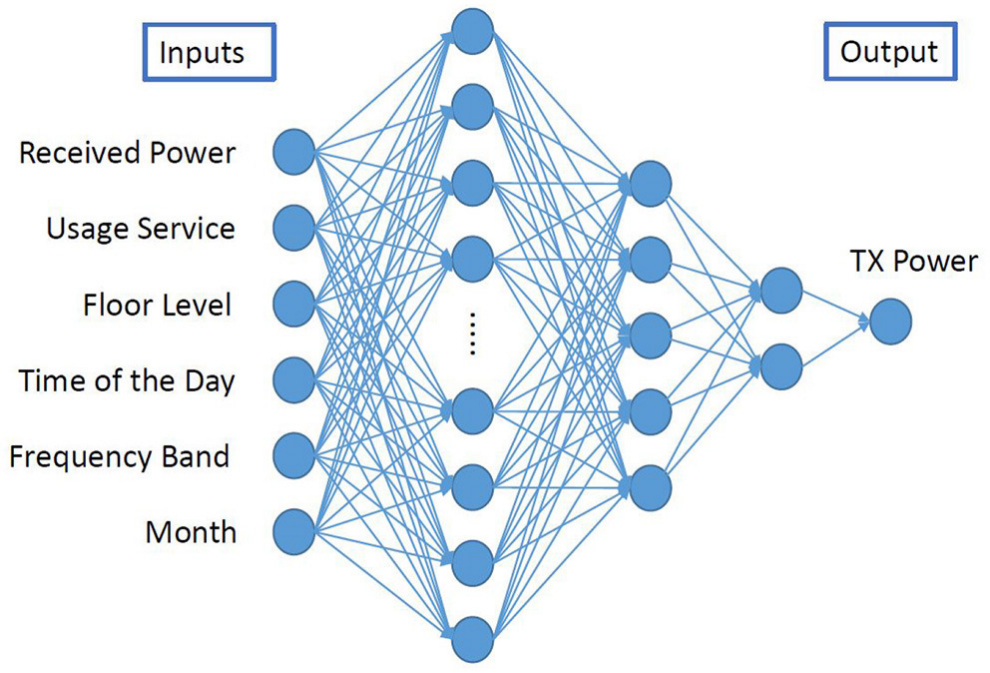
Artificial neural network structure.

A classic feed-forward neural network is built to predict UL TX power in this work. The detailed network structure can be found in Figure 2. The input layer takes six features related to DL network connections and information from the measurement environment. Then three fully connected layers with decreasing number of neurons (10, 5, and 2) are followed after the input layer. The hyper-parameters of ANN used in the current paper, including the number of layers and neurons, are determined according to grid search methods. An exponential learning rate decay scheduler is adopted to help the optimization. A large initial learning rate can speed up the training and prevent the model from being trapped in local minima. However, it may cause high oscillation in minimizing the loss function. On the other hand, a small learning rate makes the ANN model converge slower and may end up with local minima. Therefore, a reducing learning rate scheduler is applied, in order to help the ANN model converge fast and smoothly.

### 3.1. Inputs of ANN

The inputs of the ANN with their typical values and significance are shown in Table 1. According to section 2.1, the UE TX power depends on the DL path loss, which is estimated by the UE based on RSRP and/or RSSI. Consequently, RSRP and RSSI are preferred to be used as input for the prediction of the TX power. While RSRP is a parameter that could be available from off-the-shelf commercial devices (e.g., android-based applications such as XMobiSense), RSSI is not always available and it depends on the version of the android operating system (24). Therefore, in order to address the impact of the RSSI on the UL TX power estimation, we decided to build two ANN models with different combinations of inputs, either RSRP or both RSRP and RSSI as inputs while keeping all the other parameters from Table 1 unchanged.

**Table 1:** Input parameters of ANN.

**Input parameter**	**Value**	**Influence and significance**
RSRP (dBm)	[−140,−44]	Signal quality, path loss distance to base station
RSSI (dBm)	[−113, −51]	Signal quality, path loss, interference distance to base station
Usage service	WhatsApp voice call WhatsApp Video call Data upload	Amount and rate of data
Floor level	0, 1, 2, 3, 4, 5, 6	Antenna elevation angle, environment
Time of the day	Morning, noon, and afternoon	Base station traffic load, environment
Frequency band (MHz)	1,800, 2,100, 2,600	Environment
Month	May, August	Base station traffic load, environment

According to Equation (1), the UE TX power also depends on the number of resource blocks *M*, which is not accessible via android-based applications. However, the dependence of *M* on the UE usage service implies the selection of the latter parameter as an alternative input to the ANN. The other parameters from Table 1 are the features that can represent an unique property of measurement. The input “floor level” also reflects information about the path loss and the antenna elevation angle. The string values are transformed according to their type to numerical values that can be processed by the ANN. For example, the input parameter “time of the day” includes morning, noon, and afternoon that are transformed into 0, 1, and 2, respectively.

### 3.2. Assessment of Prediction Accuracy

The performance of ANN is evaluated by three metrics, MAE, root mean squared error (RMSE), and R-Squared (R^2^). Both MAE and RMSE are computed in dB. Here, smaller values of MAE and RMSE indicate more accurate predictions. R^2^ measures how close the ground truth and predictions are in terms of statistical distribution. While the perfect prediction would result in an R^2^ value of 1, the most poor fitting results in an R^2^ value approaching 0. R^2^ is defined as follows:


(2) $$\begin{array}{r} R^{2}=1-\frac{RSS}{TSS} \end{array}$$

where *RSS* and *TSS* represent, respectively, the residual sum of squares and the total sum of squares.

## 4. Results

### 4.1. Measurement Analysis

**Figure 3:**
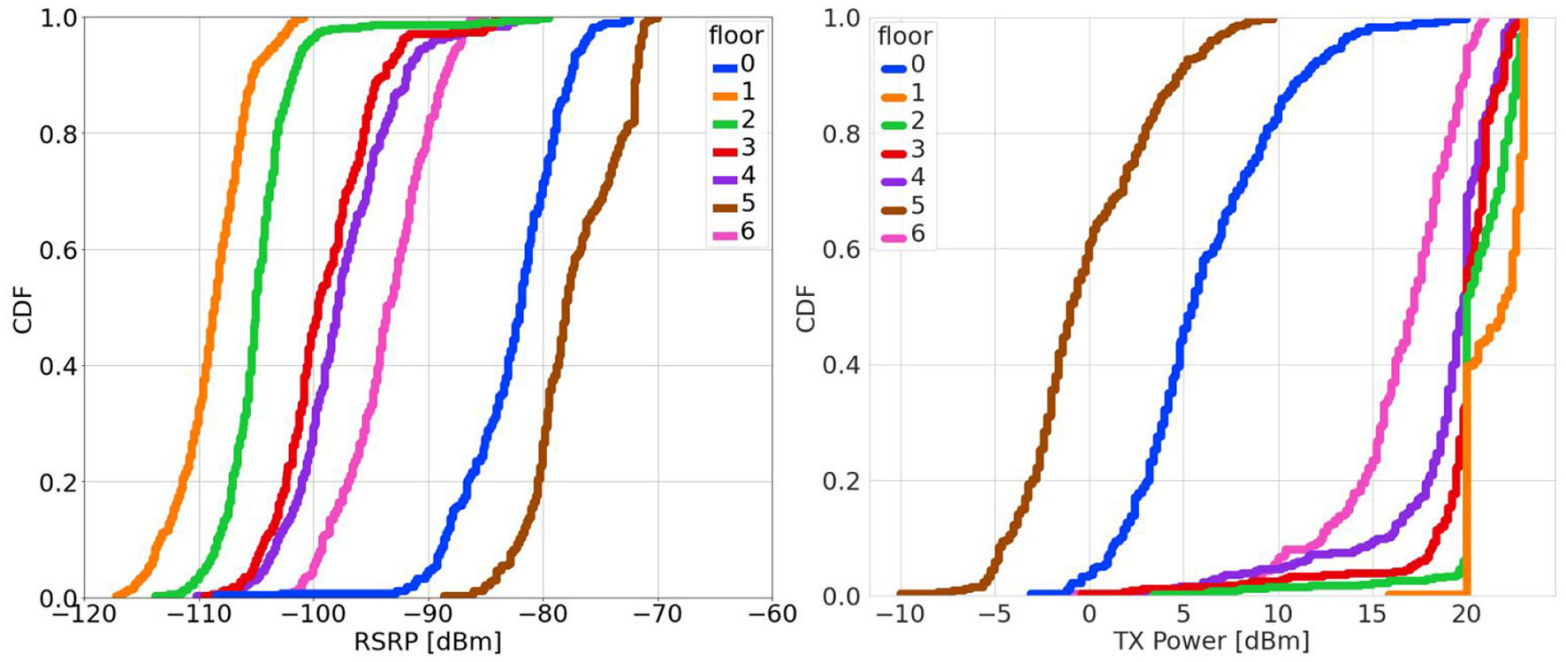
Statistical distribution of reference signal received power (RSRP) and transmit (TX) power at different floors.

Figure 3 shows the cumulative distribution function (CDF) of the RSRP and TX power variations at each floor, after aggregating all the usage services data at any time. The highest RSRP values are shown for floor 5, which means the lowest path loss and the best propagation condition. This may be due to the fact that the main gain of the active antenna base station is directed toward floor 5. The antenna gain will decrease with the antenna elevation angle and floors 1–5 have almost the same layout. This is supported by the observations in the Figure 3 that start from floor 5, except the ground floor (i.e., floor 0), decreasing or increasing the floor level will obviously yield a decrease in the RSRP values. The RSRP values on floor 6 are higher than the others due to a glass window on the roof, which permits to pass more of electromagnetic waves. Furthermore, the ground floor provides very high RSRP values, which are very close to those of floor 5. The difference in the median is less than 4 dB. This is explained by the different layout of the ground floor, where we have the entrance of the building with two glass doors. Consistent results are shown with the statistical distribution of TX powers. We note, after checking with Nemo Handy, that all the measurements occur with the same connected base station.

**Figure 4:**
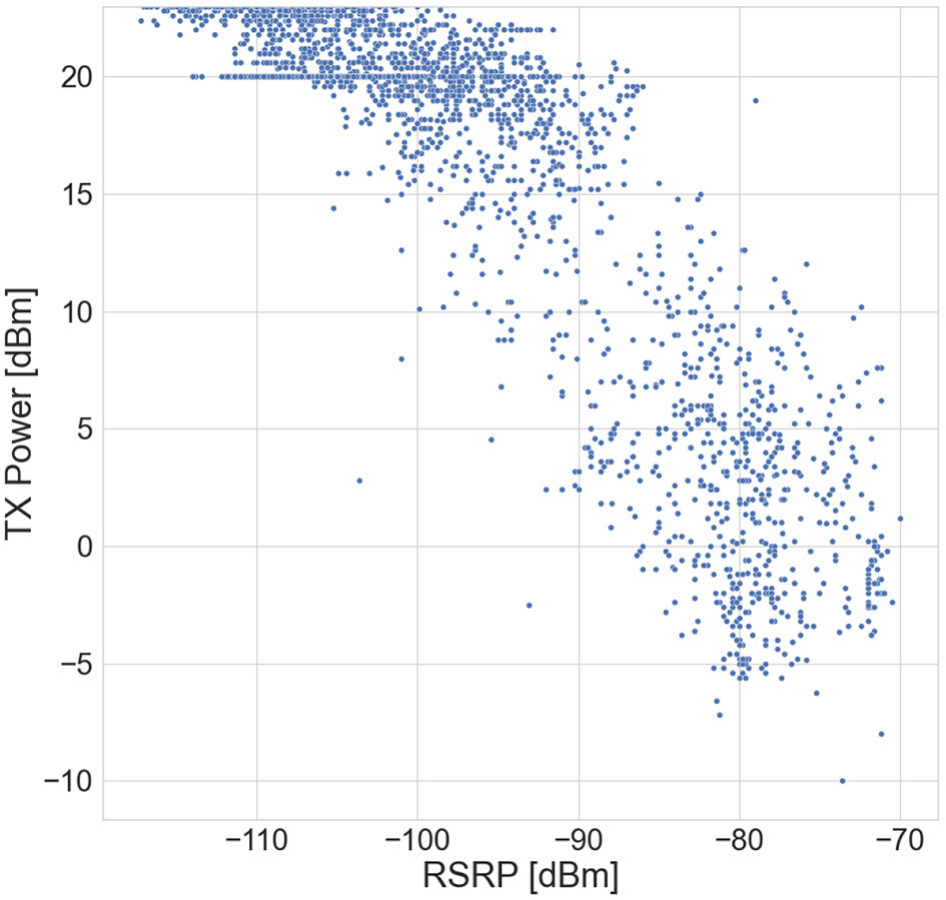
Statistical correlation between Tx power and RSRP.

The statistical correlation between the UE TX power and RSRP for all the measurement data is presented in Figure 4. Similar behavior is observed for the correlation between TX power and RSSI. Obviously, as RSRP/RSSI increases, the DL path loss decreases, and consequently TX power decreases. For each RSRP value, the variation in the TX power is due to other parameters involved in the UL power control algorithm (Equation 1), which are not accessible. However, those parameters are directly or indirectly influenced by the selected input parameters, whose correlations with TX power are shown in Table 2. We note that the Pearson correlation reflects the linear relationship between the parameters and does not reveal deeper correlations. All these reasons strengthen the requirement of the ANN model for the prediction of UE TX power, which is crucial for UL RF-EMF exposure assessment.

**Table 2:** Pearson correlation coefficient between inputs and target TX power for different averaging durations.

	**Average over 1 s**	**Average over 3 s**	**Average over 5 s**
RSRP [dBm]	−0.811	−0.865	−0.876
RSSI [dBm]	−0.793	−0.854	−0.865
Usage service	0.138	0.209	0.224
Floor level	−0.149	−0.227	−0.241
Time of the day	0.015	0.012	0.009
Frequency band	−0.184	−0.192	−0.184
Month	0.145	0.202	0.224

### 4.2. Implementation and Performance of ANN

As mentioned in section 3, the hyper-parameters of ANN are determined according to grid search methods. The neural network is trained with different potential combinations of hyper-parameters. Indeed, the optimal set of hyper-parameters with the best three-fold cross validation performance is selected. Accordingly, the hyper-parameters used in the current work are given in Table 3. In the pre-processing of measurement data, we remove the duplicates where we have the same inputs and outputs data set. Then, “RobustScaler” is used to scale the input features with outliers since it removes the median and scales the data according to the quantile range.

**Table 3:** Hyper-parameters of ANN.

**Hyper-parameters**	**Value**
learning rate	*lr* = *lr*_0_exp(−*kt*), *lr*_0_ = 0.03, *k* = 0.01
Optimizer	Adam
Activation	“elu” (hidden layers), “linear” (output layer)
Weight initializer	he_uniform
epoch	150
Batch size	48
Loss function	MSE
Train : Validation : Test	0.8 : 0.2 : 0.2

After implementing ANN, we first compare the performance from models trained with different combinations of RSSI and RSRP. Here, measurement data are averaged over 5 s and split into training and testing with the ratio of 80 and 20%. Results in Table 4 show that all three models have almost the same prediction performance, which is consistent with the strong correlation between RSSI/RSRP and TX power as shown in Table 2. Moreover, RSSI and RSRP carry redundant information. Therefore, as explained in section 3.1, only RSRP is kept as an input feature in the following model due to its easy accessibility and strong correlation.

**Table 4:** Performance comparison with different inputs.

	**RSSI**	**RSRP**	**RSSI+RSRP**
MAE [dB]	1.663	1.487	1.558
RMSE [dB]	2.501	2.365	2.394
R^2^	0.902	0.912	0.910

**Figure 5:**
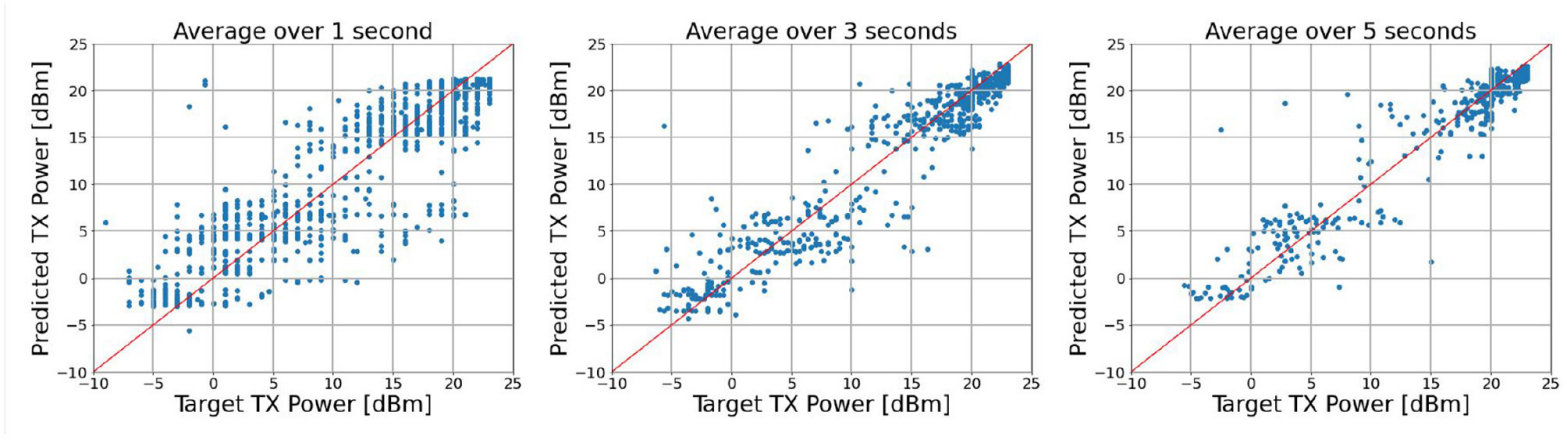
Scattering plots between predictions and true values.

Since the measurement data exported from Nemo Handy gives approximately 0–5 samples per second, we pre-process the data by computing average values over 1, 3, and 5 s. From Table 2, the correlation between inputs and target TX power is stronger with the increase of averaging duration. This implies that the corresponding prediction results in terms of MAE, RMSE, and R^2^ reveal the same improvement, as shown in Table 5. Consistently, the scattering plots in Figure 5 show that a tighter prediction is obtained with the increase of averaging duration. More clearly, the problem of vertical lines with dots from the left figure, which is caused by noise in the input data, is solved. However, we only did the averaging up to 5 s due to the limited number of measurement data (number of datasets after removal of duplicates are shown in Table 5).

**Table 5:** Performance comparison between different averaging duration.

	**Average over 1 s (8,238)**	**Average over 3 s (4,044)**	**Average over 5 s (2,510)**
MAE [dB]	2.334	1.791	1.487
RMSE [dB]	3.577	2.650	2.365
R^2^	0.831	0.897	0.912

## 5. Discussion

To the authors' knowledge, very rare works investigate building ML or ANN models based on real measurement data for the estimation of the LTE UE TX power. Similar work is done in (21) but with drive testing measurement data in outdoor environments. For the estimation of the UL TX power, they use and compare three ML methods and test different input sets. They consider uploading a file of three different sizes (i.e., 1, 3, and 5 MB) to a web server via Hypertext Transfer Protocol (HTTP), while we consider in our work more different usage services. We note that the lowest MAE in (21) is 3.166 dB with a full featured model. It raises to 4.33 dB when limiting the input features to RSRP, upload size, and velocity.

The main novelty of the current work is the exploitation of the ANN model for the LTE TX power prediction from measurement data in multi-floor indoor environments. Measurement campaigns have been carried out on a single location at each floor of a 6-floor residential building. While using several usage services (i.e., WhatsApp voice and video calls, file uploading via DropBox), data on UE TX power and input features, such as RSRP, are collected. The results are very promising with a MAE of 2.334 dB. Indeed, we believe that the proposed model has high potential in applying to more general scenarios. As shown in Table 2, UE TX power is closely dependent on the DL received power, which is RSSI or RSRP in our study. This strong correlation guarantees an acceptable prediction. So, the difficulty is to further improve the prediction performance by taking into account the varying environments. Even though measurement campaigns are very time-consuming and complicated to perform in indoor environments, more measurements are required to cover several location points on the same floor. More input features related to the floor layout, such as the number of walls, the number of windows and their penetration losses, should be considered in future work. Moreover, data should be collected over several months in order to account for the seasonality of the traffic. Last and not least, the model can include more usage services, such as voice over LTE (VoLTE).

## 6. Conclusion

The evaluation of UE TX power is very crucial for the assessment of the UL RF-EMF exposure. However, recording UE TX power requires specific equipment and carrying out measurement campaigns is complicated and time-consuming. Therefore, we aim in the present work to predict the LTE UE TX power by investigating the ANN model with easily available input features, for multi-floor indoor measurement data. First, the LTE network parameters in the indoor environment with an outdoor connected base station are collected using a specific handheld measurement device, i.e., Nemo Handy. The correlation between the DL network parameters and the target TX power are analyzed. Both DL received power indicators, RSSI and RSRP, have a strong correlation with TX power. Accordingly and since RSRP is easily available, we build a feed-forward ANN model with RSRP as input together with other parameters influencing the TX power and related to the measurement environment. Afterward, the influence of averaging duration in the data pre-processing, e.g., 1, 3, and 5 s, on the prediction accuracy is compared. The results show that averaging over 5 s for measurement data keeps a good trade-off between noise removal and a sufficient number of training data, which also has best prediction accuracy in terms of MAE, RMSE, and R^2^.

## Data Availability Statement

The raw data supporting the conclusions of this article will be made available by the authors, without undue reservation.

## Author Contributions

TM and SW conceived and wrote the manuscript. TM and MH designed and conducted the measurement campaigns. MH, SW, TM, and BA analyzed measurement and built ANN model. TM, SW, and JW reviewed the paper. All authors listed have made a substantial, direct, and intellectual contribution to the work and approved it for publication.

## Funding

The current work was partly supported by the following ANSES projects: EXPO-ENFANT (ref: 2018/RF/031) and SPUTNIC (ref: 2017/RF/017).

## Conflict of Interest

The authors declare that the research was conducted in the absence of any commercial or financial relationships that could be construed as a potential conflict of interest.

## Publisher's Note

All claims expressed in this article are solely those of the authors and do not necessarily represent those of their affiliated organizations, or those of the publisher, the editors and the reviewers. Any product that may be evaluated in this article, or claim that may be made by its manufacturer, is not guaranteed or endorsed by the publisher.
